# Dopamine in Tourette syndrome: a 30-year bibliometric analysis of hotspot evolution

**DOI:** 10.3389/fneur.2025.1589842

**Published:** 2025-09-17

**Authors:** Qian Liu, Zhiyao Zhu, Fei Luo, Yali Ding, Yuanyuan Wang, Changjiang Zhao, Bin Yuan

**Affiliations:** ^1^Department of Pediatrics, Affiliated Hospital of Nanjing University of Chinese Medicine, Nanjing, China; ^2^Department of Pediatrics, Nanjing Gaochun Traditional Chinese Medicine Hospital, Nanjing, China; ^3^Department of Pediatrics, Suqian Affiliated Hospital of Nanjing University of Chinese Medicine, Suqian, China; ^4^Department of Pediatrics, Jiangyin Hospital Affiliated to Nanjing University of Chinese Medicine, Jiangyin, China

**Keywords:** Tourette syndrome, dopamine, bibliometric, visual analysis, hotspots

## Abstract

**Introduction:**

Tourette syndrome (TS), a neurodevelopmental disorder, requires attention to the physical and psychological impacts of tics and associated comorbidities. Researchers are making efforts to clarify the pathophysiology of TS and develop effective treatments amidst its rising global prevalence. This study aimed to retrieve publications discussing TS in the context of the dopaminergic system from 1994 to 2023, summarize previous research, and analyze the general information and hotspots to provide references for future research and clinical applications.

**Methods:**

Literature was filtered from the Web of Science Core Collection. Excel, CiteSpace, VOSviewer, and Scimago Graphica were used to analyze and visualize the results.

**Results:**

A total of 482 related publications were included in the study. The United States has consistently led in research output, and Yale University demonstrates excellence in workload, impact, and collaboration. Harvey S. Singer has the highest number of publications. The hotspots include comorbidities, dopaminergic Components, candidate genes, and deep brain stimulation.

**Discussion:**

The analysis reveals that the understanding of TS is gradually evolving towards neuronal and genetic mechanisms. Concurrently, deep brain stimulation is being investigated as a treatment for refractory TS. These findings suggest a need for more in-depth research to produce higher-level evidence.

## 1 Introduction

In an article published in 1885, the French neurologist Georges Gilles de la Tourette described nine patients exhibiting involuntary movements and vocalizations, a condition that would later be named Tourette syndrome (TS) in his honor. Current estimates indicate that TS affects approximately 0.3–0.9% of children and 0.002–0.08% of adults worldwide (1). These prevalence rates highlight TS as a condition of growing public health concern. TS is a chronic neurodevelopmental disorder whose core symptoms include motor and phonic tics (1). Motor tics involve sudden, involuntary contractions of muscles in the face, neck, shoulders, or limbs, while phonic tics result from contractions of muscles in the oral, nasal, or throat regions that produce sounds. Tics are typically sudden, rapid, repetitive, non-rhythmic, and can be suppressed only temporarily. These symptoms significantly impact patients’ quality of life and social functioning. Moreover, TS is frequently associated with comorbid psychiatric conditions such as obsessive-compulsive disorder (OCD), attention deficit hyperactivity disorder (ADHD), anxiety, and depression (2).

Dopamine (DA) is a central neurotransmitter regulating a variety of critical brain functions. Dysregulation of dopaminergic signaling in the central nervous system (CNS) has been implicated in several neuropsychiatric disorders. In particular, the dopaminergic system remains the most extensively studied and clinically validated target in TS to date, an emphasis supported by both pathophysiology and treatment evidence. The established central role of dopamine in TS has spawned multifaceted, and often fragmented, pathophysiological hypotheses, encompassing diverse brain circuits, dopaminergic components (transporters, receptors, enzymes), and interacting neurotransmitter systems. To integrate this scattered landscape of cross-level complexity, a bibliometric analysis was conducted to quantitatively map global research trends. By synthesizing three decades of evidence, our study aims to delineate the evolving knowledge architecture, identify latent collaborative opportunities, thereby providing a data-driven framework to guide future research.

Bibliometrics emerged as a scientific discipline in 1969 and has since been applied extensively across various research fields (3). It employs quantitative methods to analyze scholarly publications, enabling the identification of knowledge structures, evolutionary patterns, and emerging trends within a discipline. However, to date, no bibliometric study has systematically explored the dopaminergic mechanisms underlying TS. To address this gap, this study collects and analyzes relevant literature from 1994 onward and uses computational tools to visually represent the research landscape. The aim is to identify current research hotspots, uncover underexplored issues, and provide guidance for future investigations, thereby contributing to advancements in the field of TS.

## 2 Methods

### 2.1 Data collection

**Figure 1:**
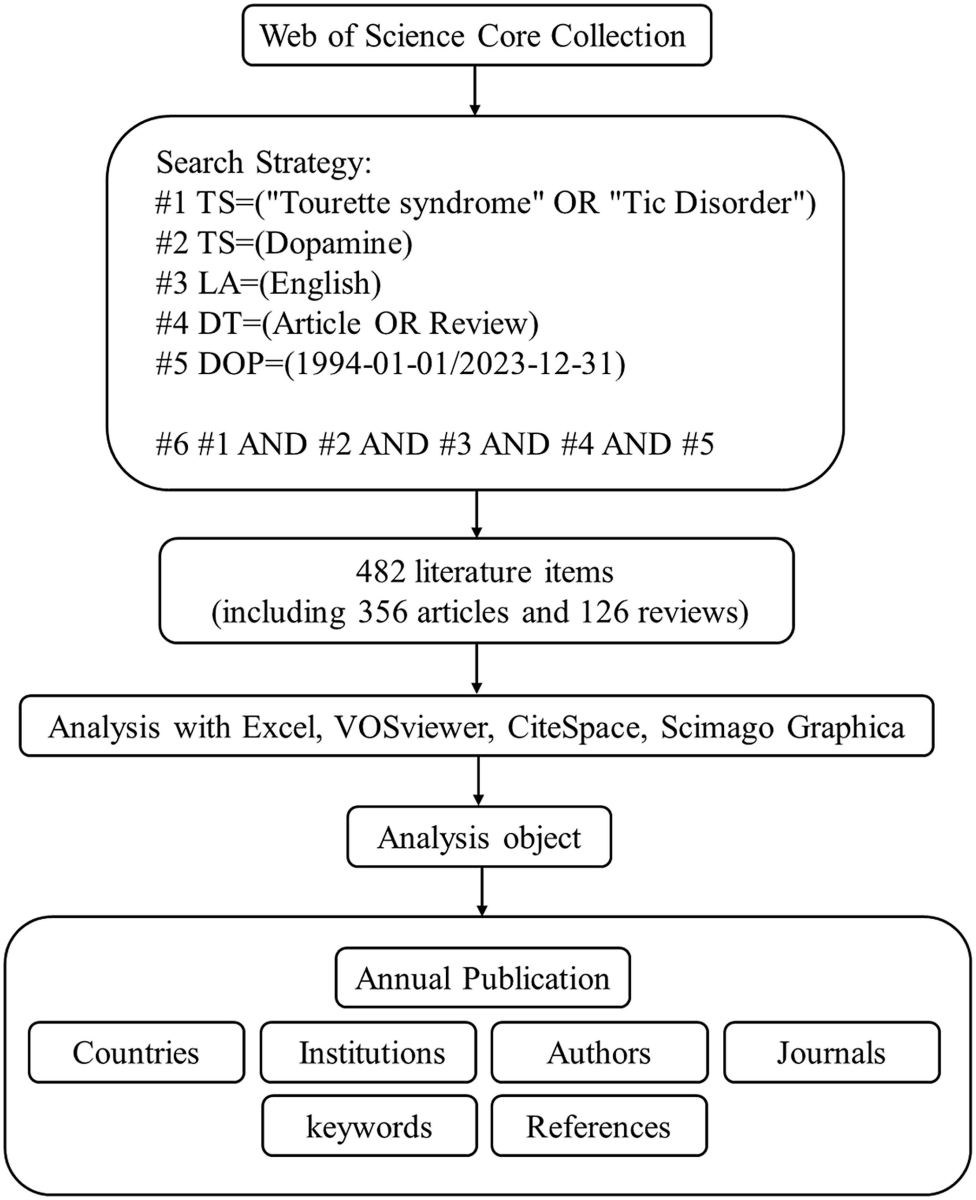
Flowchart of the search.

The Web of Science (WOS) database is widely recognized as an authoritative and rigorously curated scientific platform, offering comprehensive and regularly updated content that is highly suitable for bibliometric analysis (4). To ensure data authority and breadth of coverage, this study utilized the Web of Science Core Collection (WOSCC) as the primary source (5). The literature search was meticulously initiated on October 18, 2024. The search strategy was input in the advanced search bar as (TS = (“Tourette syndrome” OR “Tic Disorder”) AND TS = (Dopamine)) AND DT = (Article OR Review) AND LA = (English), with the time range limited from January 1, 1994, to December 31, 2023. The literature screening and data extraction processes were conducted independently by two authors. A third author adjudicated discrepancies to ensure consensus and methodological rigor. Ultimately, 482 non-duplicate valid documents were acquired. The full record and references of the 482 documents were saved in plain text file format. Figure 1 provides a detailed description of the search process.

### 2.2 Data analysis

Microsoft Excel 2019, CiteSpace 6.1.6 (6), VOSviewer 1.6.20.0 (7), and Scimago Graphica (8) were utilized for data analysis and visualization, as these tools complement each other’s strengths in handling different aspects of bibliometric analysis. Excel was employed for statistical calculations and the creation of data tables. CiteSpace, a Java-based application developed by Dr. Chaomei Chen at Drexel University (9), was instrumental in conducting time-sliced network analyses. In this study, CiteSpace was configured with time slices spanning from 1994 to 2023 (one-year intervals), using appropriate linking, selection criteria, and pruning methods. It was used to examine the distribution relationships among countries, institutions, authors, and journals, as well as the co-occurrence of keywords. VOSviewer, which employs probabilistic normalization techniques, was applied to generate and visualize bibliometric networks in various formats, including network, overlay, and density maps (7).

### 2.3 Quality control

To ensure the highest standards of methodological rigor and reporting transparency in our bibliometric analysis, this study was conducted and reported in accordance with the Reporting and Measurement of Items for Bibliometric Studies (RAMIBS) guidelines (10). This checklist provides a comprehensive framework for bibliometric research, and we adhered to its recommendations regarding data sourcing, processing, analysis, and interpretation.

## 3 Results

The 482 papers in this study, from 2010 authors affiliated with 668 organizations in 40 countries, appeared in 220 journals and cited 21,671 references from 3,355 journals.

### 3.1 Annual publication trends

**Figure 2:**
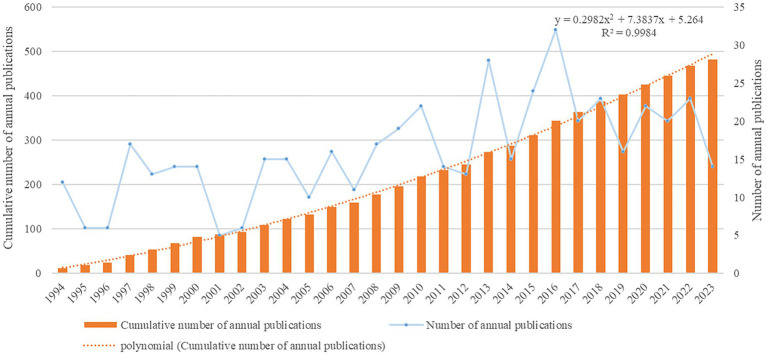
Trend of publications in the field.

From 1994 to 2023, 482 documents have been published, comprising 356 articles and 126 reviews. In Figure 2, it can be observed that before 1997, research was in its infancy, then there was a slight fluctuation but an overall upward trend. Notable increases compared to the previous year were observed in 1997, 2003, 2013, peaking in 2016 (*n* = 32). To further clarify the publication growth trend, we established a polynomial fit curve, yielding the equation *Y* = 0.2982*X*^2^ + 7.3837*X* + 5.264 (*R*^2^ = 0.9984), indicating a strong correlation.

### 3.2 Contribution of countries and institutions

**Figure 3:**
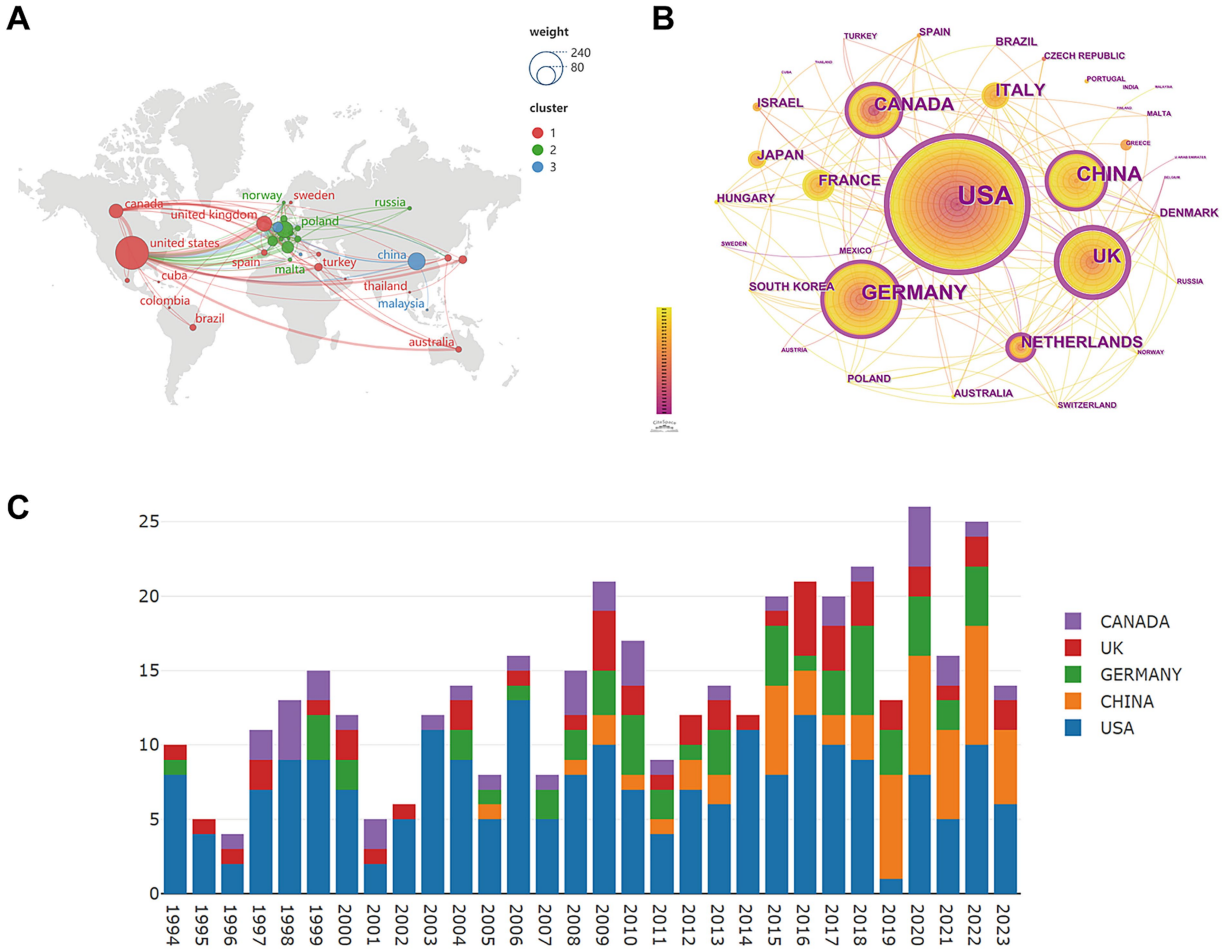
Analysis of the countries. **(A)** Geographical distribution and cooperation of global by Scimago Graphica. **(B)** Links and cooperation between countries by CiteSpace. **(C)** The yearly output of the top five publishing nations.

This study assessed publications from 668 organizations across 40 countries to identify leading countries and institutions in the field. As shown in Figure 3A, the countries involved in the research are widely distributed and are divided into three groups. The United States, leading in the field, collaborates with numerous countries across the Americas, Europe, Asia, and Australia. The other two collaborative clusters are primarily centered in Asia and Europe.

In Figure 3B, certain purple-outlined countries are more prominent than others, including USA, UK, Germany, Netherlands, Canada, and China. The magnitude of a node’s centrality, which signifies its importance, is represented by the thickness of the purple ring. A centrality value above 0.1 denotes a node’s prominent position. Table 1 lists the top 10 countries that play a crucial role.

**Table 1:** The top 10 countries with the highest centrality and number of publications.

Rank	Country	Centrality	Country	Count
1	USA	0.47	USA	221
2	UK	0.43	China	59
3	Germany	0.15	Germany	55
4	Netherlands	0.15	UK	47
5	Canada	0.11	Canada	39
6	China	0.1	Italy	29
7	Italy	0.04	Netherlands	21
8	Denmark	0.03	France	19
9	France	0.03	Japan	13
10	South Korea	0.02	Israel	12

Figure 3C presents that while the United States has consistently led, China’s output has increased over the past decade, occasionally exceeding that of the USA. Germany and the UK have also shown potential in recent years.

**Figure 4:**
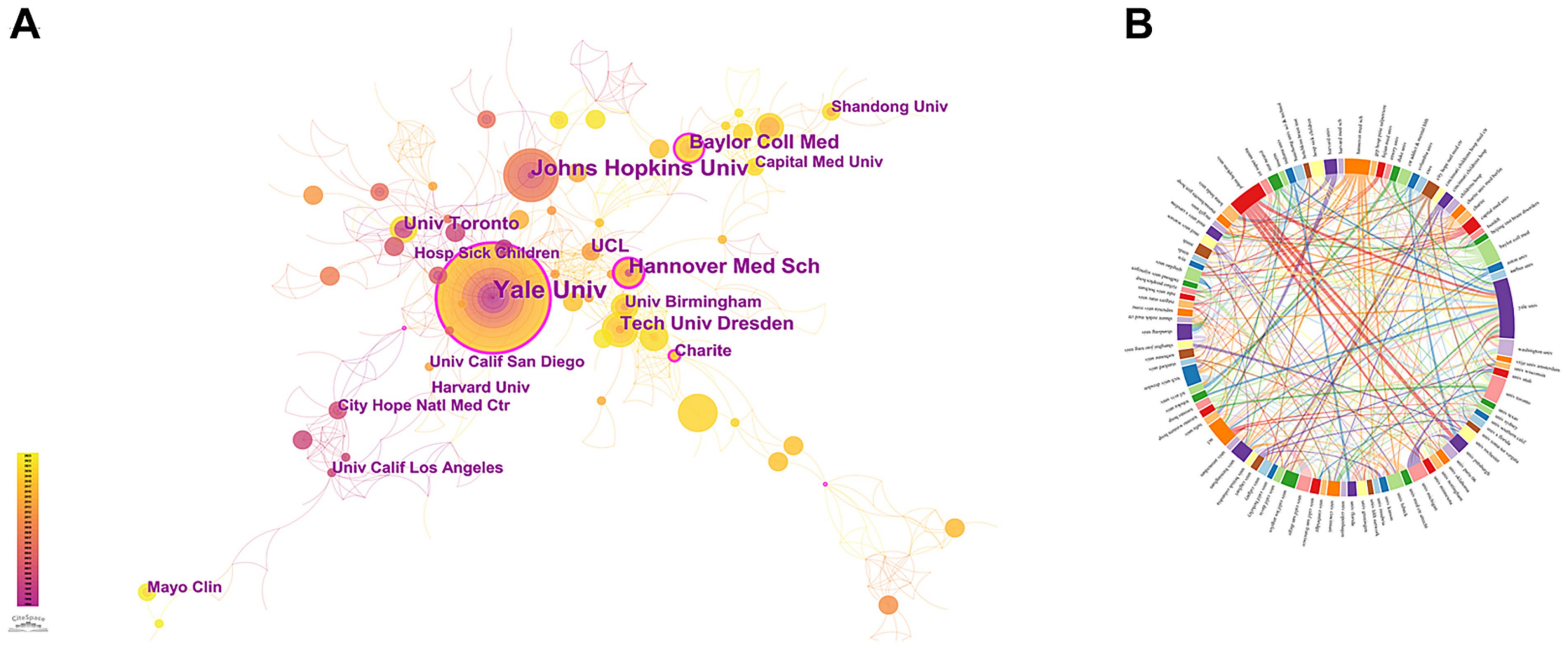
Analysis of the institutions. **(A)** Links and cooperation between institutions by CiteSpace. **(B)** Contribution and cooperation of different institutions by chord chart.

Table 2 lists the top five institutions by centrality and publication volume, which are highlighted in Figures 4A,B displays the collaborative relationships. The top five institutions are all from the USA and Germany. Yale University, which produced the highest number of publications and achieved the greatest centrality, was ranked first and maintains collaborative relationships with numerous institutions.

**Table 2:** The top five institutions with the highest centrality and number of publications.

Rank	Institutions	Centrality	Country	Institutions	Count	Country
1	Yale University	0.39	USA	Yale University	28	USA
2	Hannover Medical School	0.21	Germany	Johns Hopkins University	19	USA
3	Charité – Universitätsmedizin Berlin	0.15	Germany	Hannover Medical School	15	Germany
4	New York University	0.12	USA	Baylor College of Medicine	13	USA
5	Baylor College of Medicine	0.11	USA	Technical University of Dresden	11	Germany

### 3.3 Outstanding authors and cited authors

**Figure 5:**
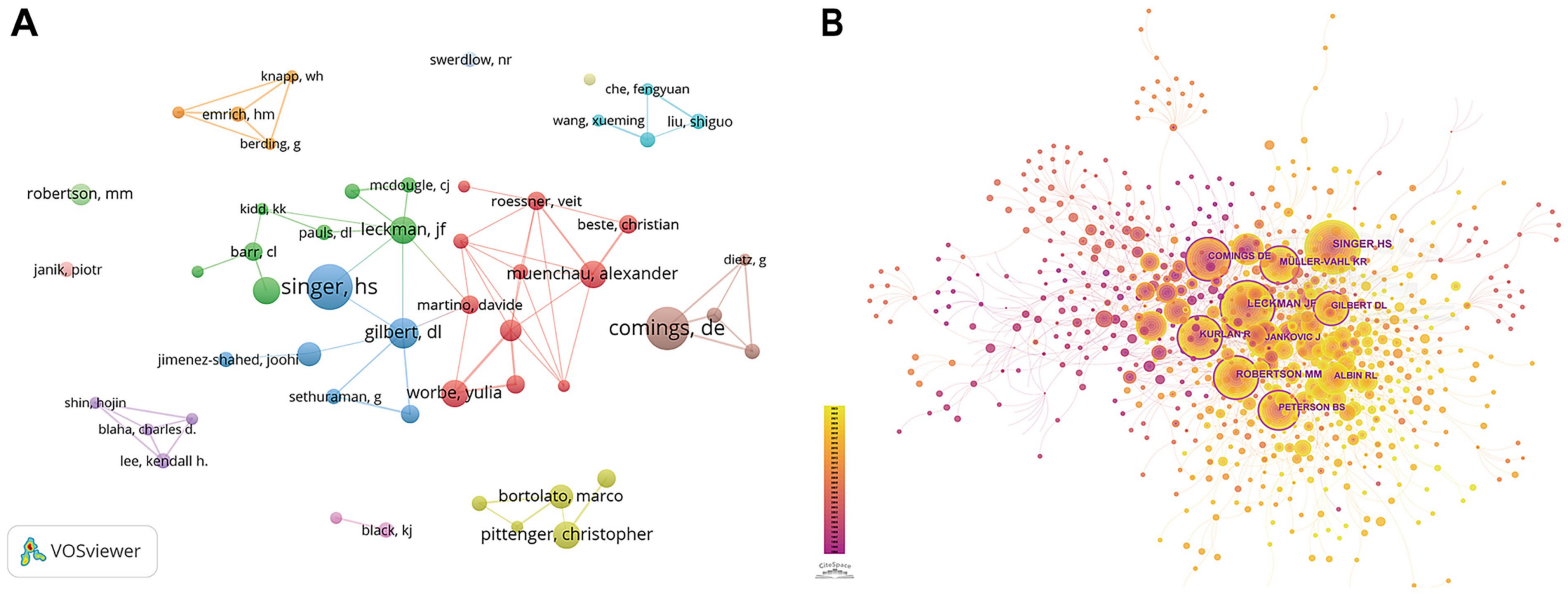
Analysis of the authors. **(A)** Visualization of authors. **(B)** Links and cooperation between cited authors by CiteSpace.

Two thousand and ten authors participated in the publications. In Table 3, Harvey S. Singer published the most, totaling 15. Following him are David E. Comings and Donald L. Gilbert, who published 14 and 10, respectively. However, it is noteworthy that, as shown in Figure 5A, apart from clusters centered around Singer, Gilbert, Leckman, and Martino, there are few connections between other groups.

**Table 3:** The top 10 most publication authors.

Rank	Author	Publications	Institutions
1	Harvey S. Singer	15	Johns Hopkins University
2	David E. Comings	14	City of Hope National Medical Center
3	Donald L. Gilbert	10	University of Cincinnati
4	Muenchau, Alexander	9	University Hospital Medical Center
5	Worbe, Yulia	9	Sorbonne University
6	James F. Leckman	9	Yale University
7	Pittenger, Christopher	9	Yale University
8	Sandor, Paul	9	University of Toronto
9	Bortolato, Marco	8	University of Utah
10	Jankovic, Joseph	8	Baylor College of Medicine

Co-citation analysis refers to the relationship between two authors when they are cited together by a third author or paper (11). Table 4 displays the top 10 cited authors by Centrality, with those having a centrality of 0.1 or higher being highlighted in Figure 5B. David E. Comings, Donald L. Gilbert, and Bradley S. Peterson are the top three authors and James F. Leckman has the most citations.

**Table 4:** The top 10 cited authors with the highest centrality.

Rank	Cited-author	Centrality	Frequency	Institution
1	David E. Comings	0.13	98	City of Hope National Medical Center
2	Donald L. Gilbert	0.13	79	University of Cincinnati
3	Bradley S. Peterson	0.13	77	University of Southern California
4	James F. Leckman	0.12	188	Yale University
5	Mary M. Robertson	0.12	133	University College London
6	Kurlan, Roger	0.12	94	Cognitive and Research Center of New Jersey
7	Kirsten R. Mueller-Vahl	0.11	91	Hannover Medical School
8	Harvey S. Singer	0.09	183	Johns Hopkins University
9	Francisco X. Castellanos	0.09	45	New York University
10	Roger L. Albin	0.08	89	University of Michigan

### 3.4 Distribution of journals and cited journals

**Figure 6:**
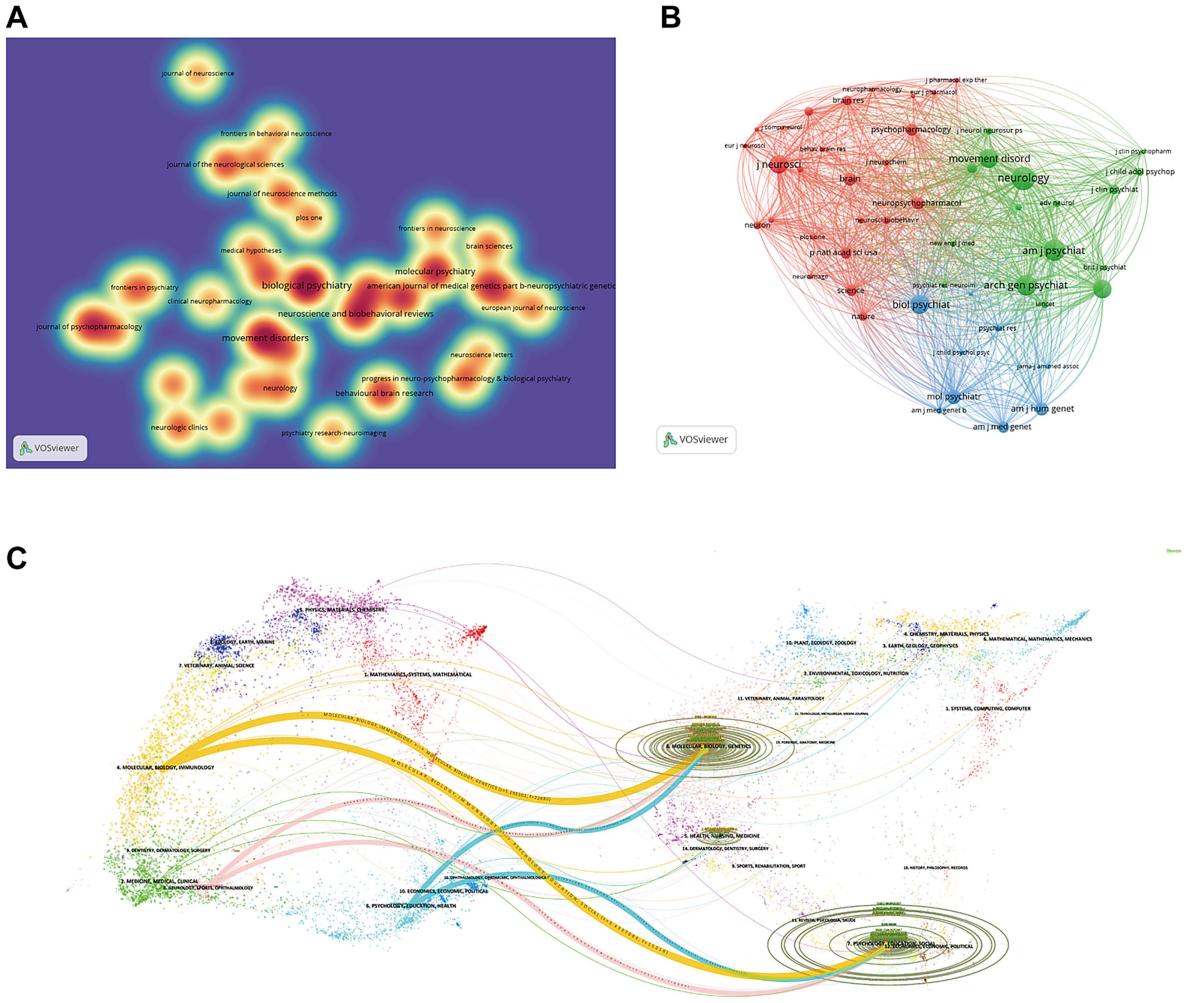
Analysis of the journals. **(A)** The weight of different publications in the density map. **(B)** Visualization of cited journals. **(C)** Dual-map overlay of journals.

In Table 5, we observe that 60% of top 10 journals are in the Q1 JCR region, 50% have an Impact factor (IF) greater than 5, and 40% have published more than 10 related articles. *Biological Psychiatry* ranks first, which makes it prominent in Figure 6A. Among these journals, *Brain* boasts the highest IF. The map of cited journals, as shown in Figure 6B, is divided into three clusters.

**Table 5:** Top 10 journals by number of publications.

Rank	Journal	Publications	IF	JCR
1	Biological Psychiatry	15	9.6	Q1
2	Movement Disorders	13	7.4	Q1
3	Molecular Psychiatry	10	9.6	Q1
4	Neuroscience and Biobehavioral Reviews	10	7.6	Q1
5	American Journal Of Medical Genetics	9	1.7	Q3
6	Advances in the Neurochemistry and Neuropharmacology of Tourette Syndrome	8	4.28	Q2
7	American Journal of Medical Genetics Part B-Neuropsychiatric Genetics	8	1.6	Q3
8	Behavioral Brain Research	8	2.6	Q2
9	Brain	7	11.9	Q1
10	Journal of the Neurological Sciences	7	3.7	Q1

The dual-map overlay technique in journal mapping reflects the disciplinary mobility at the journal level (12). Figure 6C shows that fields of molecular/biology/genetics and psychology/education/social serve as the knowledge base for the frontier research areas of molecular/biology/immunology, psychology/education/health, and neurology/sports/ophthalmology.

### 3.5 Occurrence of keywords

**Figure 7:**
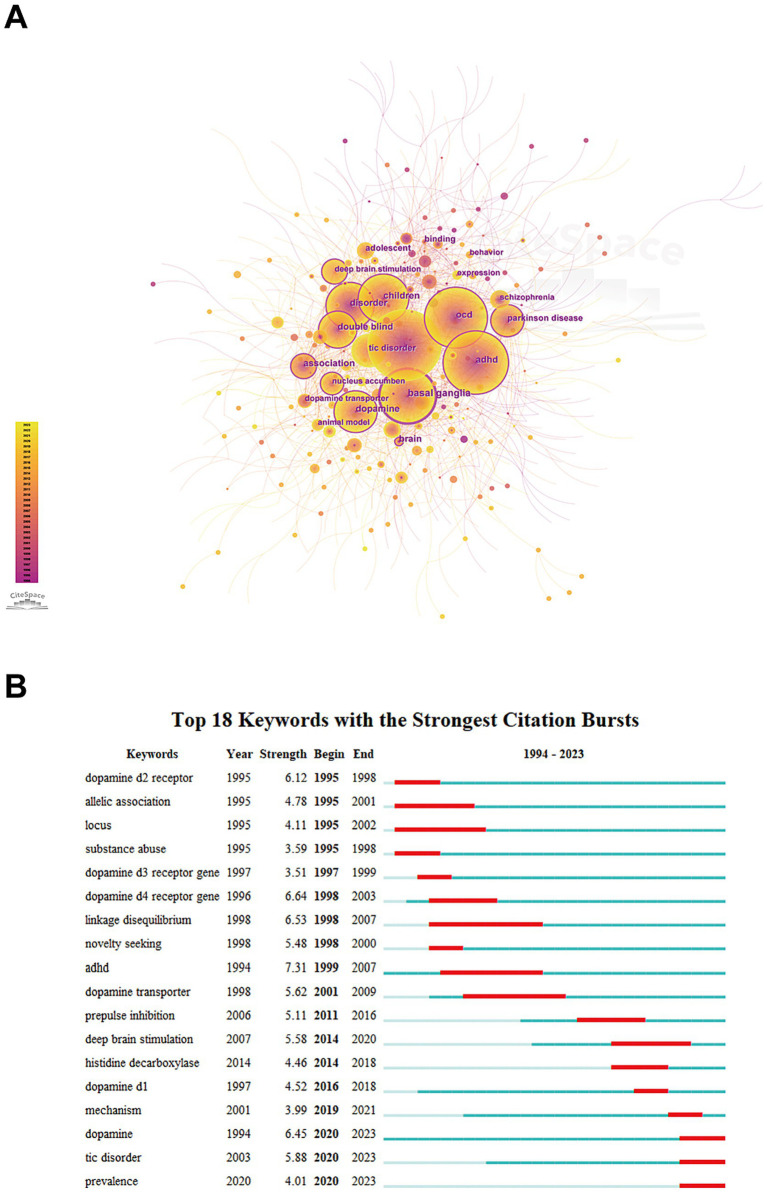
Analysis of the keywords by CiteSpace. **(A)** Links and cooperation between keywords. **(B)** Top 18 representative burst keywords.

In this study, a total of 639 keywords were identified, as shown in Figure 7A. Table 6 presents the top 10 keywords with the highest frequency and centrality. These include “Tourette syndrome,” “OCD,” “ADHD,” “Parkinson disease,” and “Schizophrenia” which represent neuropsychiatric disorders. Keywords such as “Basal ganglia,” “dopamine,” “linkage disequilibrium,” “brain,” “nucleus accumbens,” and “dopamine transporter” are associated with mechanistic aspects of TS. Additionally, “children,” “adolescent,” “double blind,” “behavior,” and “deep brain stimulation” relate to clinical trials and treatment strategies.

**Table 6:** The top 10 keywords with the highest frequency and centrality of occurrence.

Rank	Keyword	Occurrences	Keyword	Centrality
1	Tourette syndrome	327	basal ganglia	0.23
2	OCD	117	disorder	0.19
3	ADHD	111	brain	0.19
4	basal ganglia	89	ADHD	0.18
5	children	85	children	0.17
6	dopamine	70	dopamine	0.16
7	disorder	52	OCD	0.12
8	tic disorder	50	double blind	0.12
9	double blind	44	Parkinson disease	0.12
10	deep brain stimulation	39	association	0.11

A burst keyword refers to the phenomenon where any keyword appears frequently within a specific time period. This information reveals the evolution and trends of hotspots over time. In Figure 7B, the red lines indicate the times when the usage of these keywords suddenly increases. “ADHD” has the strongest citation burst, and “linkage disequilibrium” has the longest burst duration. Notably, since 2020, “prevalence” has emerged as a new burst term and has continued to the present.

### 3.6 Co-cited representative literature

**Figure 8:**
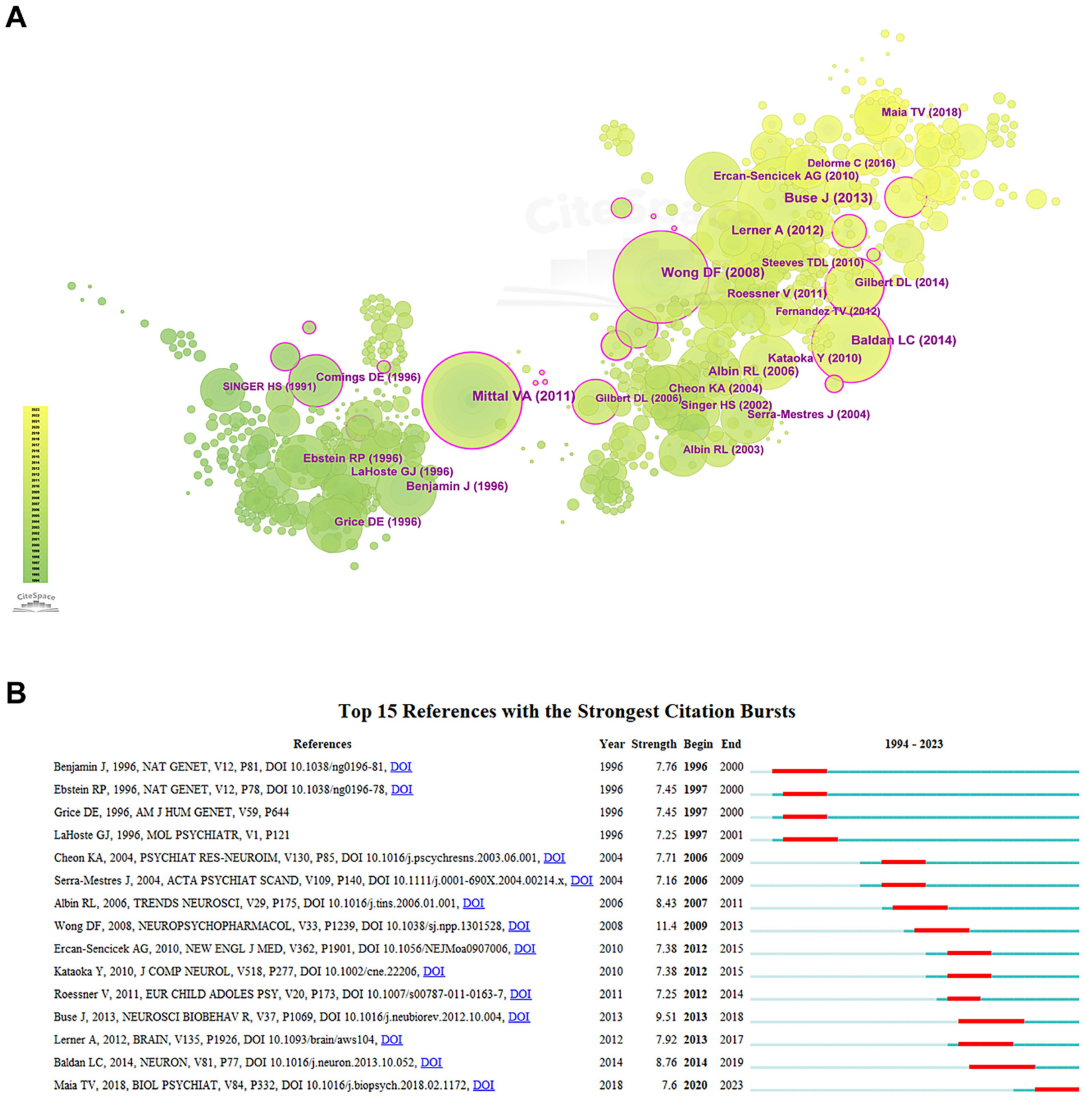
Analysis of the references. **(A)** Links and cooperation between references. **(B)** The top 15 references with the highest citation burst values.

The cited references are displayed in Figure 8A and Table 7 provides details of the top 10 articles. The article published by Vijay A. Mittal in 2011 ranks first in citation count. In Figure 8B, the work “*Mechanisms of dopaminergic and serotonergic neurotransmission in Tourette syndrome: clues from an in vivo neurochemistry study with PET*” by Dean F. Wong exhibits the highest citation strength. Additionally, “*Dopaminergic disturbances in Tourette syndrome: an integrative account*” by Tiago V. Maia, which has shown a notable citation burst since 2020, provides an in-depth discussion on hypotheses of TS and is regarded as a foundational reference in the new era.

**Table 7:** The top 10 references by the frequency of occurrence.

Rank	References	Author	Frequency
1	DOI: 10.1016/j.psychres.2011.06.006	Mittal VA	24
2	DOI: 10.1038/sj.npp.1301528	Wong DF	23
3	DOI: 10.1016/j.neubiorev.2012.10.004	Buse J	23
4	DOI: 10.1016/j.neuron.2013.10.052	Baldan LC	19
5	DOI: 10.1093/brain/aws104	Lerner A	17
6	DOI: 10.1016/j.tins.2006.01.001	Albin RL	16
7	DOI: 10.1038/ng0196-81	Benjamin J	15
8	DOI: 10.1097/WNF.0000000000000017	Gilbert DL	14
9	PMID: 9118321	LaHoste GJ	14
10	DOI: 10.1002/cne.22206	Kataoka Y	14

## 4 Discussion

As far as we know, this represents the first comprehensive bibliometric analysis mapping the research landscape of dopamine’s role in Tourette syndrome. The field has evolved from initial observations on the efficacy of dopamine antagonists in alleviating TS symptoms to the accumulation of neurobiological and molecular evidence in the 1980s and 1990s, which significantly advanced understanding of dopaminergic dysfunction in TS and guided the development of pharmacological treatment strategies. However, the rising prevalence of TS, along with challenges such as treatment-resistant cases and frequent comorbidities, has prompted a shift towards deeper genetic investigations. Concurrently, emerging technologies have created new possibilities for non-pharmacological interventions.

By synthesizing global research trends, our study uncovers the dynamic trajectory of TS research, charting its progression from fundamental mechanistic discoveries to clinical applications, while identifying key knowledge gaps and future opportunities for therapeutic development.

### 4.1 Current general information

An in-depth analysis presents a comprehensive overview of the field spanning 1994 to 2023. Keyword analysis identified research hotspots and trends.

Over the past 30 years, alongside the advancement of medical science, research on TS has been progressing, but there remains significant room for further research. In terms of countries and institutions, the United States has always been a leader, and countries like China, Germany, and the UK have also been actively developing their efforts. Yale University, demonstrating superior performance in workload, influence, and collaboration, far surpasses other organizations and holds an unshakable position in field.

In terms of authors, Harvey S. Singer, who has published the most papers, primarily researches neurosciences and psychiatry. Dr. Singer has been researching TS since the 1970s, focusing on the basal ganglia (13), dopaminergic neurotransmission (14), behavior rating scales (15), and Behavior Therapy (16). James F. Leckman, the most cited researcher, is a pioneer in TS. He is highly skilled in the diagnosis, participating in the revision of DSM-5 (17) and Yale Global Tic Severity Scale (18). He began focusing on the DA-TS link in 1986 (19), with his research mainly on animal models (20), genetic (21), and treatment (22).

Analysis of reference sources revealed that the majority of cited publications originated from high-impact scientific journals. Among these, JAMA Psychiatry, which has the highest impact factor in its category, emerged as one of the most influential journals in the field of psychiatry. These high-quality references ensure the scientific rigor and reliability of the literature survey and form a solid foundation for the research outcomes presented in this study.

### 4.2 Hotspots and frontier

Keyword analysis can reveal research hotspots and trends (12). Our analysis revealed that many high-frequency keywords correspond to comorbidities of TS. The burst keywords related to DA include “dopamine receptor,” and “dopamine transporter” reflecting ongoing investigations into how disruptions in the dopaminergic system contribute to the pathogenesis of TS. The sustained prominence of “linkage disequilibrium” as a keyword indicates a growing research focus on genetic mechanisms (23) particularly genome-wide association studies (24). Additionally “deep brain stimulation” has emerged as a prominent area of interest reflecting its evolving role as a therapeutic strategy for refractory cases.

#### 4.2.1 Comorbidities

“Isolated Tourette syndrome is the exception, not the rule” (1). 85.7% of individuals with TS had at least one psychiatric comorbidity, and 57.7% had two or more disorders (25), including ADHD, OCD, depressive disorder, anxiety disorder, bipolar disorder, schizophrenia, etc. (26). Approximately 72% of individuals with TS have ADHD or OCD as the most common comorbidities, with females more likely to have OCD and males more prone to ADHD (26). TS, ADHD, and OCD are frequently described as a “clinical triad” (27) due to their high rate of co-occurrence. These extensive comorbidities are likely attributable to overlapping neurobiological mechanisms and shared genetic vulnerabilities.

Comorbidities significantly increase the complexity of TS and complicate both diagnosis and therapeutic management. These associated conditions adversely affect learning, social functioning, and psychological development. Furthermore, individuals with comorbidities face a higher natural and unnatural mortality risk than those with isolated TS (28). Therefore, early assessment and regular reevaluation of comorbidities, accompanied by increased clinical attention and timely tailored interventions, are crucial.

#### 4.2.2 From dopaminergic components to pharmacological treatment

DA is an important catecholamine neurotransmitter in the CNS (29), playing a significant role in regulating various complex functions. Tyrosine hydroxylase (TH) and dopa decarboxylase (DDC) play key roles in the synthesis of DA. After synthesis, DA is actively transported into synaptic vesicles via the vesicular monoamine transporter 2 (VMAT2) (30), then released into the synaptic cleft through exocytosis.

DA has two modes of release, namely the tonic-phasic release (31): phasic firing, which occurs after a neuron is activated, leads to a transient release of DA, which is then rapidly removed via reuptake, forming the basis for most of DA’s functions. Tonic firing, which occurs without presynaptic input, refers to the sustained low-level release and is regarded as background activity that helps maintain stability. At this level, DA is insufficient to activate intrasynaptic dopamine receptors (DRs); instead activating extrasynaptic DRs, which inhibit phasic DA release. In synaptic cleft, DA can bind to postsynaptic receptors or dopamine terminal autoreceptors (32). Dopamine receptors (DRs) are classified into two groups based on their ability to activate or inhibit adenylate cyclase (AC): D1-like receptors and D2-like receptors. Different DRs are distributed across various brain regions, transmitting distinct signals and performing functions. After the successful transmission, the dopamine transporter (DAT) detaches DA from DRs and reuptakes it back into the presynaptic neuron (33); therefore, DAT plays a crucial role in the spatiotemporal distribution of DA in the cleft. A portion of the recycled DA is repackaged into synaptic vesicles for the next release, while another part is metabolized by monoamine oxidase (MAO), catechol-O-methyltransferase (COMT), and aldehyde dehydrogenase (ALDH) into homovanillic acid (HVA) (34), which is then excreted from the body.

Current first-line pharmacotherapies for TS, such as aripiprazole, haloperidol, risperidone, and tiapride, primarily exert their therapeutic effects through antagonism or partial agonism of the dopamine D2 receptor (35). While effective in reducing tics, this D2-centric mechanism is frequently associated with significant side effects, including extrapyramidal symptoms, sedation, and headache (35), which has motivated the pursuit of novel agents with alternative mechanisms of action. In recent years, research efforts have yielded several promising compounds that act on distinct components of the dopaminergic system. This therapeutic diversification is exemplified by the development of agents such as the D1 receptor antagonist ecopipam (36), the D3 receptor-targeting compound BL-1020 (37), and valbenazine, a selective VMAT2 inhibitor (38).

These emerging directions reflect a conceptual shift from historically predominant D2-based hypotheses toward targeting specific receptor subtypes and presynaptic regulatory proteins. This represents a pivotal advance in the neuropharmacology of TS with the goal of achieving superior efficacy alongside a more favorable tolerability profile.

#### 4.2.3 Candidate dopaminergic genes

Family, twin studies provide evidence for the strong heritability of TS (39). Candidate genes on the dopaminergic pathway primarily include DRs (DRD1, DRD2, DRD4, and DRD5), monoamine oxidase-A (MAO-A), the dopamine transporter gene (DAT1) (40).

Beyond the study of candidate genes, genome-wide association studies (GWAS) have offered novel insights into TS genetics. For instance, a large-scale GWAS of TS (41) identified several significant risk loci. The SNP rs7868992 is located within an intron of *COL27A1* and may be involved in neurodevelopmental processes. Another key variant, rs6539267, is situated on chromosome 12q23 within an intron of *POLR3B*; recessive mutations in this gene are associated with severe neurological impairment. Collectively, these findings indicate that the genetic basis of TS extends beyond a few candidate genes to encompass broader neurodevelopmental mechanisms.

#### 4.2.4 Deep brain stimulation

Deep brain stimulation (DBS) involves precise implanting electrodes in specific brain areas for continuous stimulation (42). Back in the late 1990s (43), DBS was attempted for the treatment of highly severe, drug-resistant tics (44). However, the use of this method for TS is still experimental, as there is currently uncertainty regarding best target choice. The common brain targets for TS are the globus pallidus internus (GPi) and the centromedian-parafascicular complex (CM-Pf) of the thalamus (44). The GPi, a key basal ganglia structure, regulates motor functions, and the CM-Pf complex is crucial for motor control and sensory processing. Evidence shows that CM-Pf DBS, via striatal cholinergic interneurons, triggers synaptic dopamine release and increases tonic DA levels (45).

The sustained scholarly focus on DBS underscores its value as a therapeutic option for treatment-refractory cases. However, larger multicentre studies are urgently needed to develop specific predictors of treatment response, which will be crucial to optimise patient-tailored brain target selection. Furthermore, the long-term efficacy and safety of DBS remain to be fully established to confirm its sustained therapeutic benefits and comprehensive risk profile. Simultaneously, given the ethical considerations of a brain surgery, it’s important to implement more thorough informed consent procedures. Especially for pediatric patients with developing brains, the long-term cognitive impacts of DBS are largely unknown, necessitating a careful assessment of the treatment benefits against the risks of invasive surgery and side effects. This requires a collaborative effort among ethicists, psychiatrists, and neurosurgeons.

### 4.3 Limitation

In this study, there are certain limitations. First, while TS is a multifactorial disorder involving multiple potential pathophysiological mechanisms, our analysis focused primarily on the dopaminergic system, which, although predominant, does not encompass all relevant pathways. Second, the literature was sourced exclusively from the WOSCC and limited to English-language articles published between 1994 and 2023, which may not fully represent the entire origin and the latest developments. Although these limitations do not invalidate the overall findings or the robustness of the bibliometric analysis, achieving large-scale, cross-platform integration and analysis of diverse literature sources remains an important objective for future research.

## 5 Conclusion

There are few studies that have employed bibliometric analysis to examine the temporal evolution, spatial distribution, and trend hotspots in the field of TS. This study combines bibliometric and visual analytic techniques to map the knowledge landscape of dopamine-related research in TS over the past three decades. Although the absolute number of publications remains modest, the steady increase in annual publications reflects sustained research interest and anticipates future expansion. Future efforts should focus on enhancing regional and international collaboration to overcome the limitations inherent in single-institution studies and promote the implementation of large-scale, multicenter clinical trials. At the same time, rapidly evolving scientific and technological tools should be fully leveraged to facilitate the generation of more robust and clinically impactful research.

## Data Availability

The original contributions presented in the study are included in the article/supplementary material, further inquiries can be directed to the corresponding authors.
